# Evaluating the therapeutic impact of Compound Polymyxin B Ointment on postoperative wound healing in patients with perianal abscesses

**DOI:** 10.3389/fmed.2024.1496086

**Published:** 2024-12-17

**Authors:** Yan-Zhu Li, Fu-Rong Zhou, Xiao-Juan Chen, Yong-Gan Liu

**Affiliations:** Department of Anorectal, The People’s Hospital of Zhongshan, Zhongshan, Guangdong, China

**Keywords:** wound healing, Compound Polymyxin B Ointment, perianal abscesses, postoperative care, surgical complications

## Abstract

**Introduction:**

Perianal abscesses pose a considerable obstacle in the realm of postoperative wound treatment owing to their elevated susceptibility to infection and associated consequences. Polymyxin B Ointment, a compound renowned for its antibacterial qualities, has the potential to provide therapeutic advantages by promoting wound healing and mitigating postoperative problems.

**Methods:**

Our institution conducted a thorough retrospective analysis spanning from December 2020 to December 2023 to assess the effectiveness of Compound Polymyxin B Ointment in the management of surgical wounds in patients diagnosed with perianal abscesses. The research encompassed a cohort of 100 individuals, who were classified into two groups: a control group that received conventional postoperative care, and an observation group that received supplementary treatment with Compound Polymyxin B Ointment. The evaluation of clinical outcomes involved measuring wound healing effectiveness, pain intensity using the Visual Analogue Scale (VAS), tissue swelling, exudation, necrotic tissue shedding time, duration of hospital stays, and rate of reduction in wound area.

**Results:**

The group that received Compound Polymyxin B Ointment had significant enhancements in wound healing, as seen by a noteworthy 46% of participants completing complete healing, in contrast to the control group’s 32%. The VAS was used to quantify pain levels, and the observation group reported a substantial reduction of almost 50% in ratings. Furthermore, this cohort exhibited a 45% decrease in edema and a 50% decline in exudation rates, in addition to a 50% acceleration in the shedding of necrotic tissue. The duration of the hospital stay was reduced by 40%, and the reduction in wound area was 18% higher, suggesting a more effective healing process. In addition, it is worth noting that the observation group had a lower incidence of problems, so underscoring the effectiveness of the ointment in facilitating wound healing and mitigating postoperative difficulties.

**Discussion:**

The utilization of Compound Polymyxin B Ointment as an adjuvant measure in the surgical treatment of perianal abscesses has been found to have a substantial positive impact on wound healing, pain relief, and complication reduction. This finding provides evidence for the possibility of the ointment as a helpful inclusion in post-surgical wound care procedures among this specific group of patients.

## 1 Introduction

Perianal abscess is a prevalent surgical condition characterized by localized infection and pus accumulation in the perianal region, often caused by obstruction and infection of anal glands (1, 2). Globally, its incidence is estimated at 12–28 cases per 100,000 individuals annually, with a higher prevalence among younger adults and men (3). In China, the burden of perianal abscess has been increasing due to dietary changes, delayed medical intervention, and the growing prevalence of antimicrobial resistance (4). Antibiotic resistance, particularly in Gram-negative pathogens, has become a critical global issue, as highlighted by the World Health Organization (WHO) and China’s CHINET surveillance reports (5, 6). Polymyxin B, a last-resort antibiotic for multidrug-resistant (MDR) infections, has faced increasing resistance trends, complicating systemic use. Locally applied Polymyxin B, however, offers a potential advantage in reducing bacterial load at the infection site while minimizing systemic exposure and the associated risk of resistance development (7–11). The condition significantly impacts patients’ quality of life, leading to pain, discomfort, and restricted daily activities (12, 13). Postoperative complications, such as wound infections, delayed healing, and recurrence, remain key challenges, increasing healthcare costs and prolonging recovery (14, 15). Standard treatment involves surgical drainage to remove pus and alleviate symptoms (16). However, postoperative wound management often relies on conventional care, including saline irrigation, debridement, and routine dressing changes. While these methods reduce immediate infection risks, they frequently fail to prevent bacterial colonization, mitigate inflammation, or promote granulation tissue formation (17–19). Consequently, wound healing is often delayed, with high rates of complications such as persistent exudation, secondary infections, and delayed tissue repair. This underscores an urgent need for innovative therapeutic strategies, particularly those targeting local infection control and resistance mitigation, to optimize postoperative outcomes for perianal abscess patients.

Topical antimicrobial agents, such as Polymyxin B, offer promising therapeutic potential in addressing the challenges of postoperative wound management in perianal abscesses. Polymyxin B is a well-known antimicrobial agent with proven efficacy against Gram-negative pathogens (20, 21). In wound care, its application has shown potential to reduce local bacterial colonization, mitigate inflammation, and promote granulation tissue formation—key steps in accelerating tissue repair. Understanding its impact on the pathophysiology of wound healing in perianal abscesses may provide deeper insights into optimizing postoperative care strategies (22). Given the rising global concern over antibiotic resistance, the use of topical agents like Polymyxin B is particularly advantageous. Unlike systemic antibiotics, topical application minimizes systemic exposure, thereby reducing the selection pressure for resistant bacterial strains (23, 24). This approach aligns with antimicrobial stewardship goals by preserving the efficacy of critical antibiotics while addressing localized infections effectively. From a clinical perspective, the integration of Compound Polymyxin B Ointment into postoperative care protocols has the potential to significantly enhance patient outcomes. Preliminary evidence suggests that its use may shorten hospital stays, reduce complication rates, and improve wound healing efficiency, thereby contributing to better patient recovery and reduced healthcare costs.

The primary aim of this study is to evaluate the therapeutic effects of Compound Polymyxin B Ointment on postoperative wound healing in patients undergoing surgical treatment for perianal abscesses. By leveraging clinical data, the study seeks to systematically assess the ointment’s efficacy in alleviating pain, accelerating wound healing, and reducing the incidence of postoperative complications, such as delayed healing and secondary infections. The study is grounded on the hypothesis that Compound Polymyxin B Ointment, as an adjuvant therapy, can significantly enhance wound healing outcomes compared to conventional postoperative care alone. It is anticipated that the ointment will exhibit superior clinical effectiveness by promoting tissue repair, minimizing local inflammation, and reducing bacterial colonization, thereby providing a safer and more efficacious alternative for managing postoperative wounds. The findings from this research are expected to substantiate the clinical utility of this treatment and contribute to the optimization of postoperative care protocols for perianal abscess patients.

## 2 Materials and methods

### 2.1 Study design

A retrospective analysis was performed at our hospital to evaluate the effectiveness of Compound Polymyxin B Ointment in improving postoperative wound healing in patients with perianal abscesses. The study spanned from December 2020 to December 2023, yielding a significant data collection for analysis. This study involved 50 patients who received routine postoperative treatment with the administration of Compound Polymyxin B Ointment in the experimental group. A control group was established, consisting of 50 patients who got identical postoperative care without the ointment at the same timeframe. This parallel-group approach preserved the comparability of the two groups, so augmenting the dependability of the results.

Informed consent was acquired from all participants and/or their legal guardians. This study received comprehensive assessment and approval from our hospital’s ethics committee, in strict compliance with applicable rules and the ethical requirements of the Declaration of Helsinki for research involving human beings. Methods were executed discreetly, with all personal identifiers eliminated to provide privacy protection.

### 2.2 Inclusion and exclusion criteria

The study comprises people clinically diagnosed with perianal abscesses, validated with clinical evaluation and imaging techniques. Eligible participants are those who have received surgical drainage for their abscesses and have given written informed consent for participation in the research. Moreover, inclusion necessitates participants’ readiness and capability to comply with the designated postoperative care regimen, including essential follow-up appointments and adherence to treatment procedures.

The study excludes persons receiving simultaneous therapy with other topical or systemic antibiotics, which may distort the study’s results. Individuals with a documented allergy or hypersensitivity to Polymyxin B or any components of the ointment are also prohibited. The exclusion criteria encompass patients with intricate clinical conditions, including fistula-in-ano, Crohn’s disease, or any immunocompromised status, such as HIV/AIDS, active chemotherapy, or extended corticosteroid usage, owing to their possible detrimental impact on wound healing.

### 2.3 Postoperative care protocol

All participants in the study received standardized postoperative care following the surgical drainage of perianal abscesses. This care included wound cleaning, where saline solution was utilized for irrigation and debridement until the washout fluid showed no significant purulent discharge or necrotic tissue, along with continuous monitoring for signs of infection or complications. This regimen aimed to promote healing, minimize discomfort, and prevent infection. In addition to standard care, the observation group received treatment with the topical application of Compound Polymyxin B Ointment. The procedure for ointment application was standardized. Patients were positioned in the lateral decubitus position to ensure optimal access to the wound site. The wound was first irrigated with saline solution to remove any debris and exudate. Subsequently, the wound surface was cleaned and disinfected using a medical cotton ball, ensuring that the area was adequately dried. A uniform layer of Compound Polymyxin B Ointment was then applied to the wound surface, carefully covering the area without impacting the surrounding healthy skin. The entire application process adhered strictly to aseptic techniques to prevent the introduction of pathogens.

### 2.4 Data collection and outcome measures

The primary outcome measures for this study involved a comparison of clinical efficacy between the two patient groups at 14 days postoperatively. The evaluation criteria for clinical outcomes were defined as follows:

(1) Healed: complete restoration of the external appearance and function of the anus, with complete wound healing.
(2) Significantly effective: post-treatment, the anal function is normal, with granulation tissue appearing healthy and a reduction in wound size of approximately 75% or more.
(3) Effective: post-treatment, the anal function remains normal, with relatively fresh granulation tissue, and the wound size reduction ranging from 50% to less than 75%.
(4) Ineffective: despite relatively fresh granulation tissue post-treatment, excessive exudation is present, and the wound size reduction is less than 50%. The overall clinical effectiveness rate was calculated as the sum of healed, significantly effective, and effective cases, divided by the total number of cases, and then multiplied by 100%.

The clinical outcomes were recorded by physicians during routine dressing changes, based on direct observations of the wound healing process.

Secondary outcome measures included a comparison of wound healing parameters between the two groups. The Visual Analogue Scale (VAS) (25) was employed to assess pain levels at 7 days postoperatively, with a total score of 10, where higher scores indicated greater pain intensity. Swelling and exudation of the wound at 3 and 7 days postoperatively, respectively, were evaluated and graded on a severity scale from 0 to 3, with higher scores indicating more severe swelling or exudation. Additional parameters recorded included the time taken for necrotic tissue to slough off, duration of hospital stay, and wound area measurements. The reduction in wound area was calculated using the formula: (Preoperative wound area - Wound area at 7 days postoperatively) / Preoperative wound area × 100%.

Furthermore, the incidence of complications during the follow-up period, such as delayed wound healing, anal fistula, infection, difficulty in defecation, and anal incontinence, as well as recurrence rates, were compared between the two groups.

All evaluations, including the clinical efficacy and VAS assessments, were performed by experienced surgeons with at least 6 years of clinical practice. These assessments were conducted following standardized protocols and rigorous training to ensure consistency and reliability.

### 2.5 Statistical analysis

This study employed SPSS software, Version 27.0, for comprehensive statistical evaluations. Initially, data sets were classified into quantitative or categorical kinds. The distribution patterns of these data sets were established by normality testing. Independent sample t-tests were employed to evaluate differences between groups for quantitative variables following a normal distribution, with findings presented as means ± standard deviations. In contrast, for quantitative variables that do not conform to a normal distribution, data were expressed as medians and interquartile ranges [Median (Q1, Q3)], with inter-group comparisons conducted using the Mann–Whitney U test. Categorical variables were characterized by frequencies and percentages. The Chi-square (χ2) test was utilized to examine the correlations or independence among these categorical variables. This study employed a two-tailed methodology for hypothesis testing, establishing the significance threshold at a *p*-value of <0.05 to ascertain statistical significance.

## 3 Results

### 3.1 Patient selection and allocation

**Figure 1:**
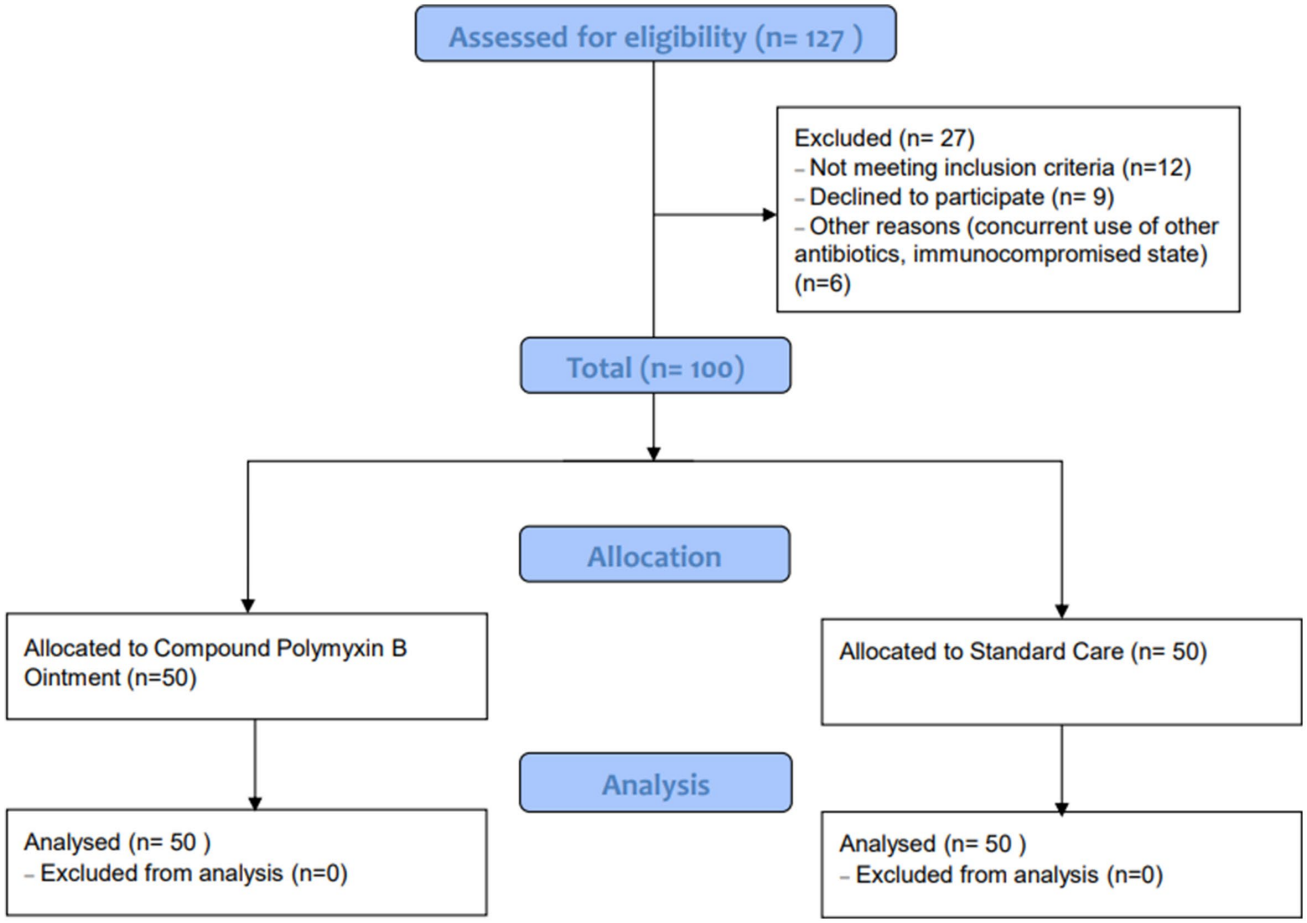
Flow diagram of the study design and population.

A total of 127 patients were assessed for eligibility, of which 27 were excluded due to various reasons: not meeting inclusion criteria (*n* = 12), declining to participate (*n* = 9), and other reasons such as concurrent use of other antibiotics or being in an immunocompromised state (*n* = 6). Ultimately, 100 patients were included in the study, with 50 allocated to the Observation group (Compound Polymyxin B Ointment) and 50 to the Control group (Standard Care). All patients in both groups were included in the final analysis. The detailed patient selection process is illustrated in Figure 1.

### 3.2 Sample size and power analysis

Then we conducted a *post hoc* power analysis. For the continuous variable, VAS scores, the calculated effect size (Cohen’s d) was 5.73. Using a significance level of 0.05 and a desired power of 0.8, the estimated sample size required was 10 patients per group. For the categorical outcome of clinical efficacy, the chi-square test indicated a required sample size of approximately 22 patients per group. Our study included 50 patients per group, exceeding these requirements and providing sufficient statistical power to detect significant differences. This ensures the robustness and reliability of our findings, mitigating concerns of sample size-related biases.

### 3.3 Patient demographics and clinical characteristics

In the control group, there were 28 male and 22 female patients, with ages ranging from 22 to 63 years, and a mean age of (43.78 ± 6.17) years. The duration of the condition varied from 5 to 11 days, with a mean of (8.13 ± 1.89) days. The locations of the abscesses included: perianal subcutaneous (23 cases), ischiorectal (5 cases), pelvic rectal (18 cases), and other sites (4 cases). The wound area ranged from 6 to 17 cm^2^, with a mean of (12.13 ± 2.39) cm^2^. In the observation group, there were 26 male and 24 female patients, with ages ranging from 25 to 64 years, and a mean age of (44.52 ± 6.48) years. The duration of the condition varied from 4 to 12 days, with a mean of (8.35 ± 1.74) days. The locations of the abscesses included: perianal subcutaneous (21 cases), ischiorectal (7 cases), pelvic rectal (17 cases), and other sites (5 cases). The wound area ranged from 7 to 18 cm^2^, with a mean of (12.42 ± 2.51) cm^2^. No significant differences were observed in the general data between the two groups (*p* > 0.05), indicating comparability.

### 3.4 Clinical efficacy in postoperative wound healing

**Figure 2:**
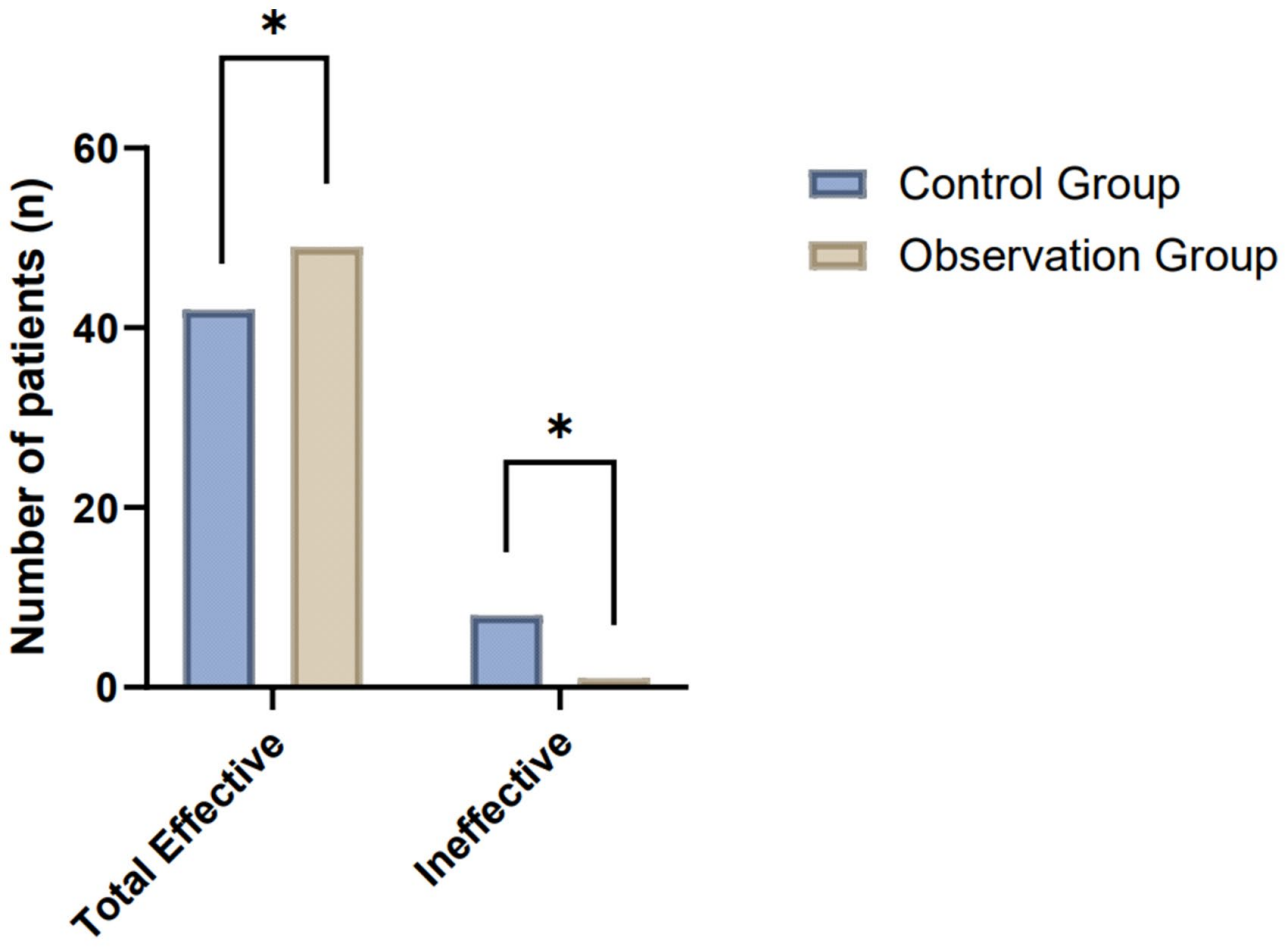
Comparative evaluation of postoperative wound healing efficacy between the control and observation groups.

The comparative analysis of clinical efficacy between the control and observation groups revealed significant differences in postoperative wound healing outcomes. In the study, both groups consisted of 50 patients each, undergoing evaluation for their wound healing progress post-treatment. The observation group demonstrated superior results, with a higher incidence of complete wound healing and overall effectiveness compared to the control group. Specifically, the observation group achieved complete wound healing in 23 patients, which was notably higher than the 16 patients in the control group. When considering the broader spectrum of positive outcomes, including complete wound healing, significantly effective, and effective categories, the observation group exhibited an overall effectiveness rate in 49 out of 50 patients. This contrasts with the control group, where 42 out of 50 patients were categorized within the effective spectrum. These results highlight the enhanced therapeutic impact in the observation group, suggesting the potential benefits of the applied treatment regimen in promoting more effective postoperative wound healing (Table 1; Figure 2).

**Table 1:** Comparative analysis of clinical efficacy between control and observation groups in postoperative wound healing.

Group	Number of patients	Complete wound healing	Significantly effective	Effective	Ineffective	Total effective
Control	50	16 (32%)	15 (30%)	11 (22%)	8 (16%)	42 (84%)
Observation	50	23 (46%)	16 (32%)	10 (20%)	1 (2%)	49 (98%)
*χ*2 Value						5.98
*p*-value						0.01

### 3.5 Results of wound healing parameters between control and observation groups

**Figure 3:**
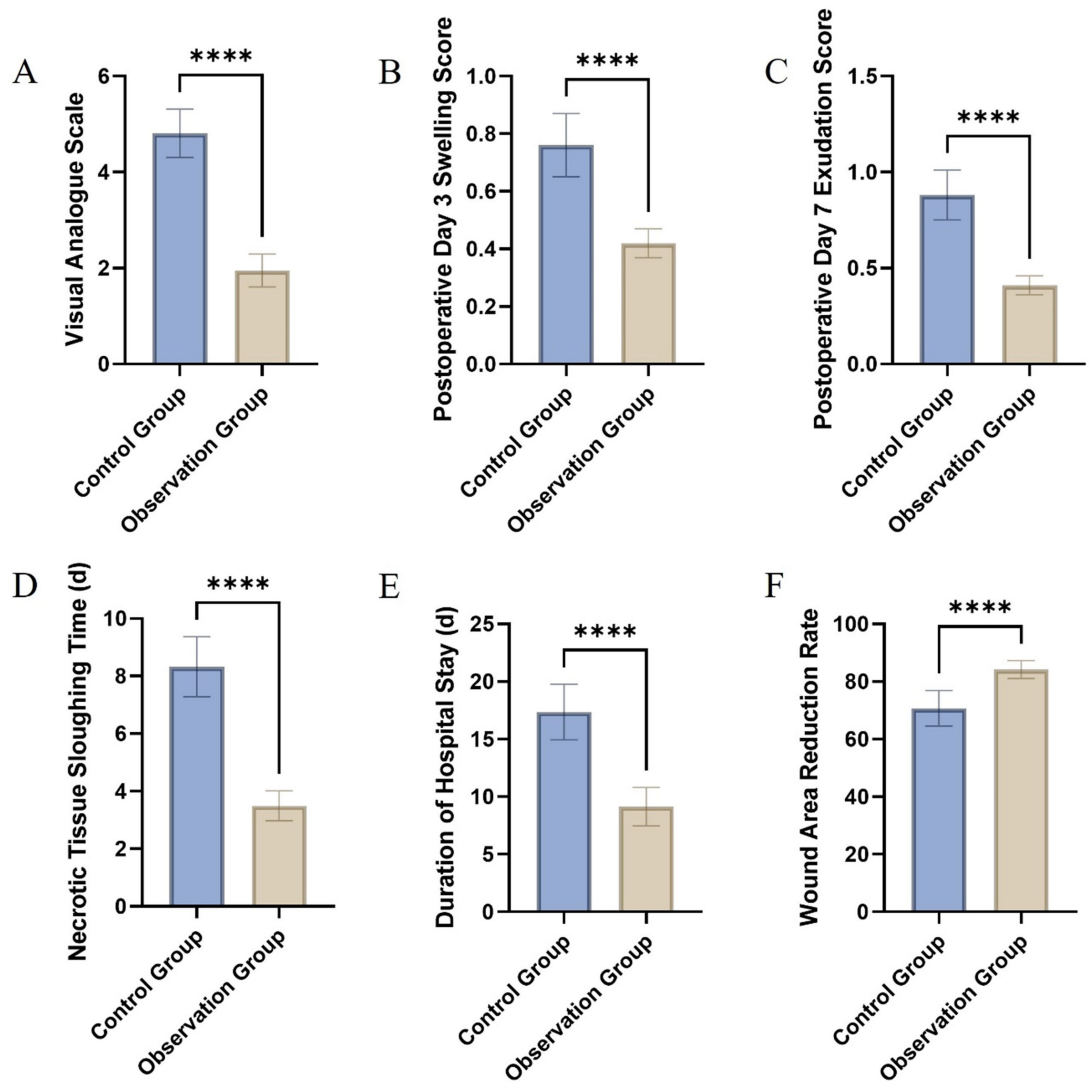
Comparative assessment of wound healing parameters between the control and observation groups: Visual Analogue Scale **(A)**, swelling score on postoperative day 3 **(B)**, exudation score on postoperative day 7 **(C)**, time to necrotic tissue sloughing **(D)**, length of hospital stay **(E)**, and wound area reduction rate on day 7 **(F)**.

The comparative evaluation of wound healing parameters between the control and observation groups yielded significant findings, elucidating the therapeutic benefits of the intervention under study. The analysis encompassed several key metrics: pain assessment through the VAS, swelling and exudation scores on postoperative days 3 and 7, respectively, the time required for necrotic tissue sloughing, the duration of hospital stay, and the rate of wound area reduction by day 7 post-surgery. A marked reduction in VAS scores was observed in the observation group, indicating significantly lower pain levels compared to the control group (Figure 3A). This difference highlights the potential analgesic effect of the treatment employed in the observation group. Similarly, swelling and exudation scores were substantially lower in the observation group on postoperative days 3 and 7, suggesting an enhanced anti-inflammatory response and better management of wound exudates (Figures 3B,C).

The time for necrotic tissue to slough off was notably shorter in the observation group, indicating a more rapid clearance of devitalized tissue, which is a crucial step in the wound healing process (Figure 3D). This accelerated clearance could contribute to a reduced risk of infection and faster overall healing. Correspondingly, patients in the observation group experienced a significantly shorter hospital stay, underscoring the potential for the treatment to facilitate quicker recovery and discharge readiness (Figure 3E). Furthermore, the wound area reduction rate by postoperative day 7 was significantly higher in the observation group, demonstrating a more efficient wound contraction and closure process (Figure 3F). This finding is indicative of enhanced reparative mechanisms, possibly attributed to the therapeutic intervention (Table 2).

**Table 2:** Comparative analysis of wound healing parameters between control and observation groups.

Group	VAS	Postoperative day 3 swelling score	Postoperative day 7 exudation score	Necrotic tissue sloughing time (days)	Duration of hospital stay (days)	Wound area reduction rate at day 7 (%)
Control	4.81 ± 0.50	0.76 ± 0.11	0.88 ± 0.13	8.32 ± 1.04	17.35 ± 2.42	70.72 ± 6.17
Observation	1.95 ± 0.34	0.42 ± 0.05	0.41 ± 0.05	3.49 ± 0.52	9.14 ± 1.67	84.17 ± 3.14
*χ*2 Value	33.45	19.90	23.86	29.37	19.74	13.74
*p*-value	<0.01	<0.01	<0.01	<0.01	<0.01	<0.01

VAS, Visual Analogue Scale.

### 3.6 Results of complication rates between control and observation groups

**Figure 4:**
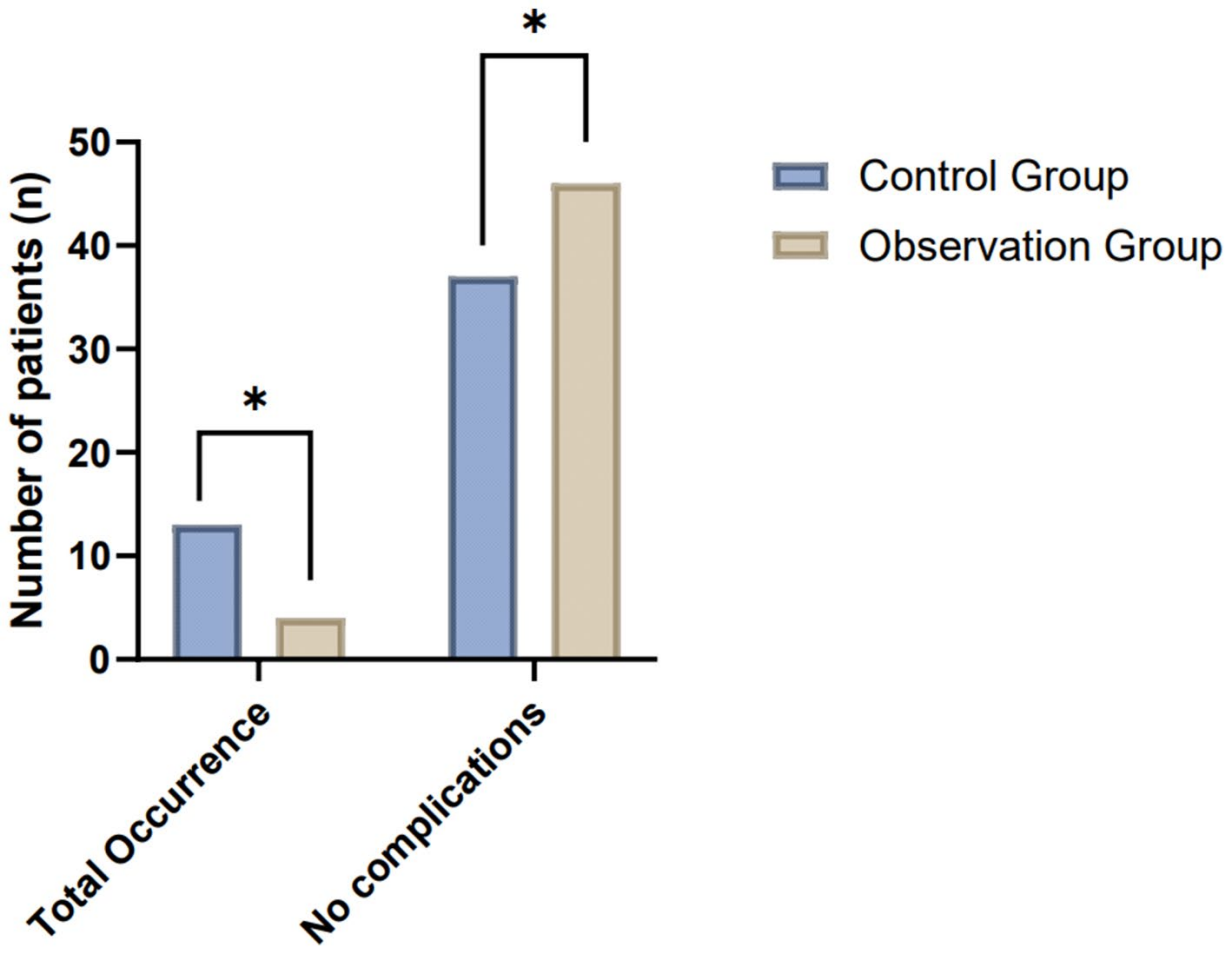
Comparative analysis of postoperative complication rates between the control and observation groups.

In assessing the post-treatment complication rates between the control and observation groups, both comprising 50 patients each, a notable variance in outcomes was observed. This analysis encapsulated several complications typically associated with the treatment of the condition under study, including delayed healing, formation of anal fistulas, infections, defecation difficulties, and anal incontinence. The observation group exhibited a markedly lower incidence of all listed complications, suggesting an enhanced recovery profile. Specifically, instances of delayed healing and infection were significantly reduced, with only 2 and 1 cases reported in the observation group, respectively, compared to 3 and 4 in the control group. Moreover, the observation group showed a complete absence of anal fistula and anal incontinence complications, underscoring the potential efficacy of the treatment approach in mitigating these specific postoperative concerns. The cumulative occurrence of complications further highlights the disparity, with the observation group experiencing a total of 4 instances compared to 13 in the control group. This significant reduction in the total occurrence of complications points to a potentially more favorable safety and efficacy profile of the therapeutic regimen employed in the observation group (Table 3; Figure 4).

**Table 3:** Comparative analysis of complication rates between control and observation groups.

Group	Number of patients	Delayed healing	Anal fistula	Infection	Defecation difficulty	Anal incontinence	Total occurrence
Control	50	3 (6%)	1 (2%)	4 (8%)	3 (6%)	2 (4%)	13 (26%)
Observation	50	2 (4%)	0 (0%)	1 (2%)	1 (2%)	0 (0%)	4 (8%)
*χ*2 Value							5.74
*p*-value							0.02

## 4 Discussion

The management of surgical wounds following perianal abscess drainage poses significant clinical challenges due to the high risk of infection and delayed healing associated with the complex microbiota and anatomical proximity to fecal matter (10, 26). This study aimed to evaluate the therapeutic effects of Compound Polymyxin B Ointment as an adjunctive treatment in improving postoperative wound healing outcomes in patients with perianal abscesses. This study highlights the potential of Compound Polymyxin B Ointment as an innovative adjuvant therapy for postoperative wound management in perianal abscess patients, addressing gaps in conventional care. Unlike standard postoperative practices, which primarily rely on saline irrigation and dressing changes, this study demonstrated that incorporating Polymyxin B was associated with enhanced clinical outcomes, including higher effectiveness rates, reduced pain, accelerated necrotic tissue clearance, and shorter hospital stays (27, 28). Additionally, the reduction in postoperative complications such as infection and delayed healing underscores the clinical relevance of this intervention. The study’s methodological rigor, including the use of standardized protocols and validated outcome measures, enhances the reliability and applicability of the findings. By effectively targeting local bacterial colonization and reducing reliance on systemic antibiotics, the use of Polymyxin B aligns with global antimicrobial stewardship efforts. These results suggest that this topical approach offers a valuable addition to postoperative care protocols, potentially improving patient recovery, reducing complication rates, and alleviating healthcare burdens, particularly in settings with limited resources or high antibiotic resistance.

The baseline characteristics of the control and observation groups were well-matched, with no significant differences in demographic or clinical parameters such as age, gender distribution, duration of the condition, abscess location, or wound area. This comparability ensures that any observed differences in clinical outcomes can be attributed to the intervention, thereby reinforcing the internal validity of the study. The inclusion of diverse abscess locations and wound sizes enhances the generalizability of our findings, making the results applicable to a broader patient population. The superior wound healing outcomes in the observation group highlight the efficacy of Compound Polymyxin B Ointment, supporting its use in promoting faster and more effective postoperative wound healing. The Table 1 provided a structured framework for categorizing wound healing outcomes, ranging from complete healing to minimal improvement. This classification, documented by experienced surgeons during routine dressing changes, ensured consistent and reliable evaluation of treatment efficacy. The VAS, an internationally recognized tool for pain assessment, complemented this by providing a simple and reliable measure of patient-reported pain levels. The routine use of VAS in clinical practice underscores its role in objectively assessing postoperative pain, thereby enhancing the overall robustness of the study’s findings. Both tools, implemented by highly trained clinicians, ensured precision and minimized observer variability, contributing to the validity of the study.

Routine microbiological cultures were not performed in the absence of overt signs of infection, such as increased exudation, redness, swelling, or systemic symptoms like fever. This approach aligns with clinical guidelines, which recommend cultures primarily when infection is clinically suspected. Conducting unnecessary cultures could lead to inefficient resource use and increased antibiotic overuse, potentially fostering antimicrobial resistance. The International Wound Infection Institute (IWII) (29) supports this selective approach to wound cultures, emphasizing their role in guiding appropriate antimicrobial therapy when needed. Our focus remained on evaluating the clinical efficacy of Compound Polymyxin B Ointment in enhancing wound healing and reducing complications, which was effectively monitored through direct clinical observations and outcomes.

The enhanced wound healing noted in the observation group, shown by a higher rate of complete wound closure and overall efficacy, can be predominantly attributed to the potent antibacterial activities of Polymyxin B. This chemical effectively reduces bacterial colonization and prevents infection at the lesion site, hence decreasing the inflammatory burden. Inflammation, while crucial for wound healing, can be detrimental if prolonged or severe, leading to tissue damage and impaired recovery. The proliferative phase of wound healing is likely expedited by the controlled inflammatory response facilitated by the antibacterial properties of Polymyxin B. Furthermore, the reduced swelling and exudation scores in the observation group suggest that Compound Polymyxin B Ointment may possess anti-inflammatory properties, possibly through the modulation of cytokine production or the inhibition of inflammatory mediators (30, 31). This phenomenon not only alleviates pain, as seen by the reduced VAS ratings, but also creates an optimal environment for tissue regeneration and rejuvenation.

The observation group demonstrated a significant decrease in the time required for necrotic tissue sloughing, suggesting an enhanced debridement process maybe facilitated by the ointment administration. The efficient removal of necrotic tissue is crucial to prevent microbial proliferation and promote the formation of viable granulation tissue. By maintaining a moist wound environment, the ointment enhances autolytic debridement, which accelerates the removal of devitalized tissue and supports the formation of granulation tissue. This mechanism is essential for transitioning the wound into the proliferative phase of healing and improving tissue integrity. The ointment’s ability to maintain a moist wound environment may enhance this process, as it is known to stimulate autolytic debridement and improve overall wound healing (32). The extensive therapeutic efficacy of Compound Polymyxin B Ointment is underscored by the notable decrease in the incidence of sequelae, such as delayed healing, anal fistulas, and infections, within the observation group. The possible alleviation of these issues is likely to be affected by various factors, including the antibacterial, anti-inflammatory, and wound healing properties of the ointment. Moreover, the absence of anal incontinence and fistula formation noted in the observation group highlights the ointment’s potential in preserving the functional integrity of the anorectal area. This may be ascribed to mechanisms related to tissue remodeling and fortification (33).

The 14-day observation period represents a critical timeframe for assessing early therapeutic efficacy in acute wound management. Improvements observed during this period align with the natural progression of the acute wound healing trajectory, where infection control, reduction in inflammation, and granulation tissue formation occur. By demonstrating substantial clinical benefits within this short evaluation period, the study highlights the early-phase impact of Compound Polymyxin B Ointment in optimizing postoperative wound care. The observation group demonstrated reduced hospital stays, attributable to the clinical advantages of expedited wound healing and diminished complications, along with potential economic benefits from shortened inpatient treatment duration and related healthcare costs. The multifaceted mechanisms of Polymyxin B—including antibacterial, anti-inflammatory, and tissue-regenerative properties—work synergistically to enhance wound healing outcomes. This comprehensive approach likely accounts for the notable clinical improvements observed during the study period and underscores the ointment’s therapeutic potential in perianal wound care.

This study has several limitations. The retrospective design may introduce selection bias and limit the collection of microbiological data, such as bacterial flora and antibiotic susceptibility, which could provide deeper insights into the therapeutic effects of Compound Polymyxin B Ointment. While the sample size was adequate, the single-center focus and lack of detailed patient comorbidity data may affect the generalizability of the findings. Additionally, the study was limited to evaluating standardized clinical parameters without microbiological analyses or comparisons with other wound care agents. Future research should employ prospective, randomized designs with larger, diverse populations to validate these findings. Incorporating microbial culture and sensitivity testing would enable a more comprehensive understanding of bacterial dynamics and antibiotic efficacy. Exploring the comparative effectiveness of Polymyxin B against other therapeutic agents and assessing its economic benefits in broader contexts is also recommended.

## 5 Conclusion

In conclusion, the adjunctive use of Compound Polymyxin B Ointment in postoperative care for patients with perianal abscesses could significantly alleviate pain, reduce wound swelling and exudation, and accelerate wound healing. This therapeutic approach might also effectively lower the incidence of complications, suggesting its potential as a beneficial treatment modality in the management of post-surgical wounds in this patient population.

## Data Availability

The raw data supporting the conclusions of this article will be made available by the authors, without undue reservation.
